# Supplementary material for: The English Debating Self-Efficacy Scale: Scale development, validation, and psychometric properties
Source: PLoS One. 2025 Feb 26;20(2):e0314879. doi: 10.1371/journal.pone.0314879 (PMC11864526; doi:10.1371/journal.pone.0314879)
Supplement: S1 Table — (DOCX) [file pone.0314879.s001.docx]

Appendix A: English Debating Self-efficacy Scale

DS1. I am able to construct clear and persuasive arguments.

DS2. I am able to provide effective evidence to support my position (such as reliable sources, quotes from authorities, conclusions from research papers, etc.).

DS3. I am able to identify logical errors between the opponent's arguments and evidence.

DS4. I am able to adjust debate strategies flexibly to respond to opponents.

TC1. I actively participate in team discussions, sharing my perspectives and listening to and respecting the opinions of team members.

TC2. I am capable of promptly identifying and clarifying any errors arising from the negligence of our team members.

TC3. I collaborate closely with debate team members, ensuring that our arguments support each other to form a strong overall performance.

LP1. I can comprehend the viewpoints expressed by my team members and opponents in English.

LP2. I can accurately articulate my viewpoints in English.

LP3. I can use appropriate and varied English vocabulary to make the debate more persuasive.

LP4. I can employ specific English intonation to express particular viewpoints, attitudes, or emotions.

LP5. I can utilize appropriate pauses to avoid ambiguity.

LP6. I can flexibly adjust my intonation and pace in English to adapt to different contexts and audiences.
