# Supplementary material for: The English Debating Self-Efficacy Scale: Scale development, validation, and psychometric properties
Source: PLoS One. 2025 Feb 26;20(2):e0314879. doi: 10.1371/journal.pone.0314879 (PMC11864526; doi:10.1371/journal.pone.0314879)
Supplement: Attachment — Submitted filename: Response to Editor.docx [file pone.0314879.s004.docx]

Dear Editor,

Thank you for your valuable feedback on our manuscript. We appreciate you pointing out the issues, and we have taken the necessary steps to address them. Specifically, we have rewritten and paraphrased certain sections to reduce the similarity with existing published articles.

Additionally, we have uploaded the scale developed and validated in our study as supplementary material for your review.

We are grateful for your support and guidance. Please feel free to let us know if there are any further revisions or additional information required.

Best regards,

Yanchao Yang, Ph. D.

Associate Professor
Institute of International Language Services Studies,
Macau Millennium College
Alameda Dr. Carlos D'Assumpção n.º 255, China Civil Plaza, 8º andar. Macau, China
