# Supplementary material for: The English Debating Self-Efficacy Scale: Scale development, validation, and psychometric properties
Source: PLoS One. 2025 Feb 26;20(2):e0314879. doi: 10.1371/journal.pone.0314879 (PMC11864526; doi:10.1371/journal.pone.0314879)
Supplement: Attachment — Submitted filename: Response to Reviewers.docx [file pone.0314879.s006.docx]

Dear Editor,

I am writing to express my heartfelt gratitude to you and the reviewers for your detailed and insightful comments on our manuscript. Your thoughtful feedback not only highlights the strengths of our work but also points out areas for improvement, demonstrating a deep engagement with our research. We truly appreciate the significant time and effort that you and the reviewers invested in providing such valuable insights. This constructive criticism has been instrumental in refining our study and enhancing its overall quality. We are genuinely thankful for your support and guidance throughout this process.

In response to the reviewers' thoughtful critiques, we have meticulously revised our manuscript to address each comment and suggestion. We greatly value the insights provided, which have prompted us to reevaluate and refine various aspects of our work. Our revisions include a series of adjustments aimed at enhancing both the clarity and rigor of our study. We believe these changes not only strengthen our arguments but also improve the overall coherence of the manuscript. Below, we have outlined the specific modifications made in response to each comment, as detailed in the accompanying table.

| Reviewer Comments | Reply |
| --- | --- |
| Abstract and keywords: The abstract is not well organized. Please, follows a clearer logical flow from purpose to methods, results, and conclusions. Provides a more concise overview of the study's key elements. Explicitly mentions the name of the developed scale (EDSS) early on and includes a brief conclusion to summarize the main findings. | Thank you for your suggestion. We have restructured the abstract to include the research background and highlight the research gap. Please refer to the updated manuscript for details.  In addition, we included a brief conclusion in the abstract to summarize the main findings. |
| The keywords are not appropriate for the study's topic. What is EFA; CFA? | Thank you for your suggestion. We replaced EFA and CFA with "exploratory factor analysis" and "confirmatory factor analysis," respectively, to enhance clarity and ensure that readers who may not be familiar with the abbreviations can easily understand the methods being discussed. |
| Introduction: The introduction provides a solid background on the importance of English debate, however the flow for the write up is not well organized. Also, I was surprised not to read about the gap this study wants to fill. What is the statement of problem for this research? What are the research objectives? What is the main purpose of this research? All these has to be well spelt out in an organized manner. | Thank you for your suggestion. To make the research objectives and gaps clearer to readers, we added subheadings in the introduction section. This not only enhances the readability of the text but also helps readers better grasp the key points and logical structure of the study. Through this approach, we aim to guide readers more smoothly in understanding the background and significance of the research. In addition, we also added a section on the significance of the study. Please refer to the updated manuscript for details. |
| Literature Review: The literature review has no introduction and sub-headings. Why? | Thank you for your suggestions. We have added subheadings in Literature Review section, and, in addition, included an introduction to the Literature Review section. Please refer to the updated manuscript for details. |
| Methodology: The methodology section needs to be reviewed. Here are a few suggestions to improve this manuscript:  a) Start my telling us what research design was used and research methods employed and not ethical consideration.  b) For research design, can the author(s) also consider a longitudinal study to track changes in debate self-efficacy over time, especially in relation to debate training or experience.  c) Convenience sampling is practical, it may introduce bias. The use of convenience sampling technique has to be well justified.  d) The three dimensions (Debating skills, Team collaboration, and Language proficiency) seem interesting. Why was it limited to these three. Can you consider exploring additional dimensions such as: Critical thinking, Argument construction, Rebuttal skills and even public speaking confidence.  e) For validation, please consider including test-retest reliability to assess the stability of the scale over time to strengthen your work. | (1) Thank you for your suggestions. We have adjusted the Method section by moving the ethical considerations to the end and placing the Research Design at the beginning.  (2) The research design is a cross-sectional design, and due to privacy concerns, identifiable information about students was not collected during data collection, making a longitudinal design not feasible at this stage. However, we plan to adopt a longitudinal design in future studies to gather additional evidence, such as test-retest reliability.  (3) The use of convenience sampling has already been justified in the text.  (4) Thank you for your suggestions. The dimensions of the scale were derived from interviews, and while the current research outcomes cannot be modified, we will explore the possibility of expanding the scale's dimensions in future studies.  (5) As noted earlier, the research design is cross-sectional, and we did not collect identifiable information to protect participants' privacy, rendering a longitudinal design infeasible at this time. We intend to incorporate a longitudinal design in future research to obtain further evidence, including test-retest reliability. |
| Discussion: The discussion section needs adequate citation to either argue or support your findings. | Thank you for your suggestions. We have added an explanation of the criterion-related validity in the Discussion section. Please refer to the updated manuscript for details. |
| Implications: Elaborate on how the EDSS can be used in educational settings, debate training programs, or for self-assessment by debaters. | Thank you for your suggestions. We have added a section outlining the anticipated implications of the research for academia, teaching, and practice, including for teachers, students, and debate coaches. Please refer to the updated manuscript for details. |
| Conclusion: The conclusion section needs to be expanded. | Thank you for your valuable suggestions. We have enriched the content of the Conclusion to provide a more comprehensive summary of our findings and their implications. |
| The abstract needs restructuring to clearly state the study's gap, key findings, and conclusion. | Thank you for your suggestion. We have restructured the abstract to include the research background and highlight the research gap. Please refer to the updated manuscript for details. |
| The literature review lacks structure and requires subheadings for better organization. | Thank you for your suggestion. We have added headings and subheadings to the manuscript for better organization. Additionally, we have restructured the literature review, especially highlighting the scarcity of proper scale for English debating self-efficacy. Please refer to the updated manuscript for details. |
| The introduction should be reorganized to clearly state the research gap, problem statement, objectives, and purpose. | Thank you for your suggestion. We have divided the introduction into four sections. The first section provides background, emphasizing the importance of debating. The second section highlights the significance of self-efficacy and briefly discusses the current research gap, specifically the lack of a suitable measurement scale for English debating self-efficacy. The third section focuses on the research objective, which is to develop and validate a scale for measuring English debating self-efficacy. In addition, we also added a section on the significance of the study. |
| The methodology section needs significant revision, including a clear statement of the research design and methods employed. | Thank you for your suggestion. We have added a section Research Design in the Methods section and emphasized the methods employed. Please refer to the updated manuscript for details. |
| The discussion section requires more citations to support or argue the findings. | Thank you for your suggestions. We have added an explanation of the criterion-related validity in the Discussion section. Please refer to the updated manuscript for details. |
| To strengthen the study, consider expanding the scale's dimensions, employing a longitudinal design, and including test-retest reliability. | Thank you for your suggestions. The dimensions of the scale were derived from interviews, and since the current research outcomes cannot be modified, we will explore the possibility of expanding the scale's dimensions in future studies. Additionally, to protect student information, we did not collect any identifiable information, and this study employed a cross-sectional design. As such, we did not validate longitudinal aspects such as test-retest reliability. However, we plan to design a longitudinal study in future research. Please refer to the updated manuscript for details. |
| The implications section should elaborate on the EDSS's practical applications in various settings. | Thank you for your suggestions. We have added a section outlining the anticipated implications of the research for academia, teaching, and practice, including for teachers, students, and debate coaches. Please refer to the updated manuscript for details. |
| In a sentence, the authors should state the gap in the study. The gap in the study lies in the lack of detailed analysis of the variables that may have influenced the result of absence. | Thank you for your suggestion. We have divided the introduction into four sections, among which current research gap was briefly mentioned, specifically the lack of a suitable measurement scale for English debating self-efficacy. In addition, we have also restructured the abstract to include the research background and highlight the research gap. Finally, we have also highlighted the research gap in the literature. |
| Results were absent in the abstract, and the conclusion was not well crafted. Additionally, the conclusion fails to summarize the key findings and implications of the study effectively. | Thank you for your valuable suggestions. We have made several revisions to the abstract based on your feedback. Specifically, we have included the research background and added the Cronbach's Alpha values for the three dimensions. Additionally, we have highlighted the study's structure and incorporated implications in the conclusion. We have also rewritten the abstract to enhance clarity and effectiveness. Your insights have been instrumental in improving our work. |
| The study fails to identify its design as well as the selection process.  Who are the study population? | Thank you for your suggestion. We have added a section Research Design in the Methods section and emphasized the methods employed. Please refer to the updated manuscript for details.  In addition, we have expanded the sample description section to include details about the research site and the sampling method employed. Furthermore, we have provided a description of the participants, noting that they are all university students. Additionally, we included information about the data collection timeline, the use of the survey platform, the reasons for its selection, and the data cleaning methods applied. |
| How were the items generated?  Prior to the distribution of the question, were the items validated? If yes, how was it validated? What type of validation was conducted?  Instrument How were the items generated? Before the analysis, how many items were included in the questionnaire? How many items were eliminated? What were the criteria for such elimination? Which item(s) was/were eliminated, if any? | Thank you for your suggestions. We have added detailed descriptions of the interview process and its purpose, item generation, and expert validation for content validity. Please see the updated manuscript for more information. |
| I think the ethical considerations should be the late subsection after the analytical procedure in the methodology. | Thank you for your thoughtful suggestion regarding the placement of ethical considerations. We have taken your advice and relocated this important information to the end of the methods section. |
| Some of the references are not complete. Ensure to include the doi for those that have it. | Thank you for your suggestions. We have added the DOIs for the relevant articles and updated the References section accordingly. |
| Occurrence of overlapping text | Thank you for your reminder. I have paraphrased the content with similarity issues. |
| Supporting Information File | Thank you for your reminder. We have re-uploaded the EFA and CFA data files. |

Once again, we would like to extend our heartfelt thanks to you and the reviewers for your invaluable guidance and unwavering support throughout this process. Your insights have been instrumental in shaping our work, and we genuinely appreciate the collaborative spirit that this review embodies. We eagerly look forward to your feedback on our revised submission and hope that our efforts meet your expectations.

Warm regards,
