# Supplementary material for: The English Debating Self-Efficacy Scale: Scale development, validation, and psychometric properties
Source: PLoS One. 2025 Feb 26;20(2):e0314879. doi: 10.1371/journal.pone.0314879 (PMC11864526; doi:10.1371/journal.pone.0314879)
Supplement: Attachment — Submitted filename: Reviewer Comments - PONE-D-23-27190_R2.docx [file pone.0314879.s005.docx]

The research is on the development and validation of an English Debate Efficacy Scale The author(s) needs to work on the following corrections:

1. Abstract and keywords: The abstract is not well organized. Please, follows a clearer logical flow from purpose to methods, results, and conclusions. Provides a more concise overview of the study's key elements. Explicitly mentions the name of the developed scale (EDSS) early on and includes a brief conclusion to summarize the main findings.
2. The keywords are not appropriate for the study's topic. What is EFA; CFA?
3. Introduction: The introduction provides a solid background on the importance of English debate, however the flow for the write up is not well organized. Also, I was surprised not to read about the gap this study wants to fill. What is the statement of problem for this research? What are the research objectives? What is the main purpose of this research? All these has to be well spelt out in an organized manner.
4. Literature Review: The literature review has no introduction and sub-headings. Why?
5. Methodology: The methodology section needs to be reviewed. Here are a few suggestions to improve this manuscript:
a) Start my telling us what research design was used and research methods employed and not ethical consideration.
b) For research design, can the author(s) also consider a longitudinal study to track changes in debate self-efficacy over time, especially in relation to debate training or experience.
c) Convenience sampling is practical, it may introduce bias. The use of convenience sampling technique has to be well justified.
d) The three dimensions (Debating skills, Team collaboration, and Language proficiency) seem interesting. Why was it limited to these three. Can you consider exploring additional dimensions such as: Critical thinking, Argument construction, Rebuttal skills and even public speaking confidence.
e) For validation, please consider including test-retest reliability to assess the stability of the scale over time to strengthen your work.
6. Discussion: The discussion section needs adequate citation to either argue or support your findings.
7. Implications: Elaborate on how the EDSS can be used in educational settings, debate training programs, or for self-assessment by debaters.
8. Conclusion: The conclusion section needs to be expanded.
